# Oncocytic Adrenocortical Neoplasm Diagnosed after Robot-Assisted Adrenalectomy

**DOI:** 10.1155/2015/515071

**Published:** 2015-11-17

**Authors:** Andrew C. Harbin, Andrew Chen, Siddharth Bhattacharyya, Jasvir S. Khurana, Joshua R. Kaplan, Daniel D. Eun

**Affiliations:** ^1^Department of Urology, Temple University Hospital, 3401 N Broad Street, Philadelphia, PA 19140, USA; ^2^Department of Pathology, Temple University Hospital, 3401 N Broad Street, Philadelphia, PA 19140, USA

## Abstract

Oncocytic tumors, composed of eosinophilic, mitochondria-rich cells, can occur in several locations throughout the body. These tumors can occur in the adrenal cortex and are rarely malignant. We report a case of a patient presenting with an incidental adrenal mass which was later diagnosed as a oncocytic adrenocortical neoplasm (OAN). The patient is a 53-year-old man found to have a 7.2 cm right adrenal mass, incidentally found by computed tomography (CT). After metabolic workup was negative, a right robotic adrenalectomy (RA) was performed. Pathologic analysis revealed clusters of large cells with abundant eosinophilic and granular cytoplasm, consistent with OAN. This pathology is rare, with only about 150 cases described in the literature. It occurs in females 2.5 times more frequently and more commonly on the left side. Diagnosis is usually made by imaging criteria, typically with CT or magnetic resonance imaging (MRI). Treatment is generally surgical, since OAN can be malignant in some cases. Differentiation between benign and malignant OAN is done based on the Lin-Weiss-Bisceglia criteria and can be difficult. If malignancy is diagnosed, recurrence is common and close surveillance should be performed.

## 1. Introduction

Oncocytic tumors are composed of eosinophilic cells (oncocytes) containing a high number of mitochondria. They are not specific to any organ and can occur in several locations, such as salivary glands, kidney, thyroid, parathyroid, and pituitary. Tumors occurring within these locations are for the most part benign (oncocytomas). Oncocytic neoplasms have been rarely reported in the respiratory tract and choroid plexus and are sometimes malignant [1]. The term is used for tumors with varying biological behaviors. Oncocytic tumors of adrenal cortex are rare with just over 150 cases in the literature. Further, in this location, their biological behavior cannot be predicted with certainty. We report a case of an oncocytic adrenocortical neoplasm (OAN) that was diagnosed incidentally.

## 2. Case Report

**Figure 1:**
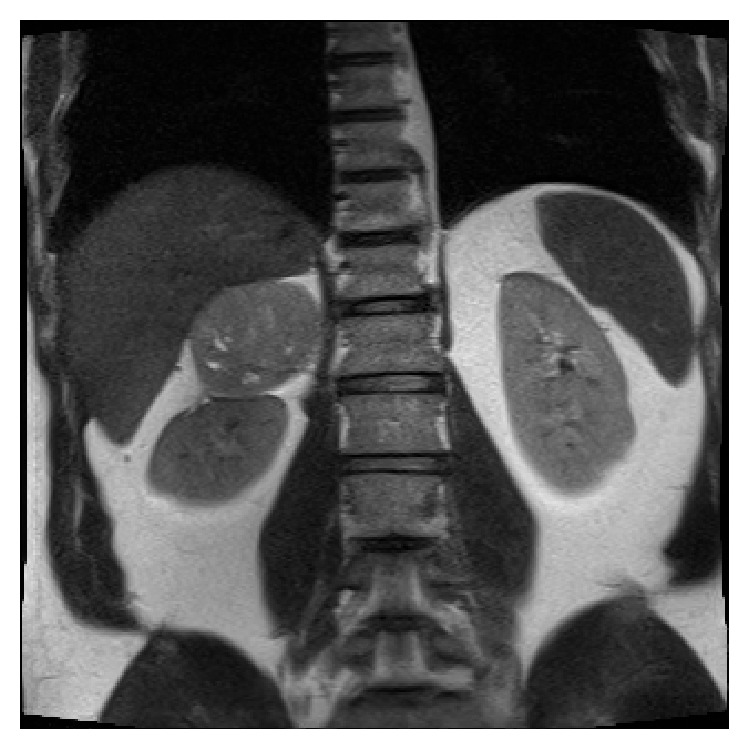
Coronal section of magnetic resonance imaging (MRI) showing 7.3 cm right adrenal mass.

The patient is a 53-year-old man originally diagnosed with an incidental finding of a large right adrenal mass. He presented with abdominal pain, for which a computed tomography (CT) of the abdomen and pelvis was performed, showing a 7.2 cm right adrenal mass. A CT scan 3 months prior demonstrated the mass to be 6.8 cm. Magnetic resonance imaging (MRI) was also performed, revealing a 7.3 cm mass with heterogeneous T2 signal and low T1 signal (Figure 1).

Serum chemistries were normal; there was no increase in cortisol or catecholamines. The patient had a prior history of prostate cancer treated with radical prostatectomy, but no other cancer history. Since the mass had a heterogeneous T2 signal on MRI and angiomyolipoma could be ruled out by the absence of fat on CT, suspicion for malignancy was high. The decision was made to perform a right robotic adrenalectomy (RA).

**Figure 2:**
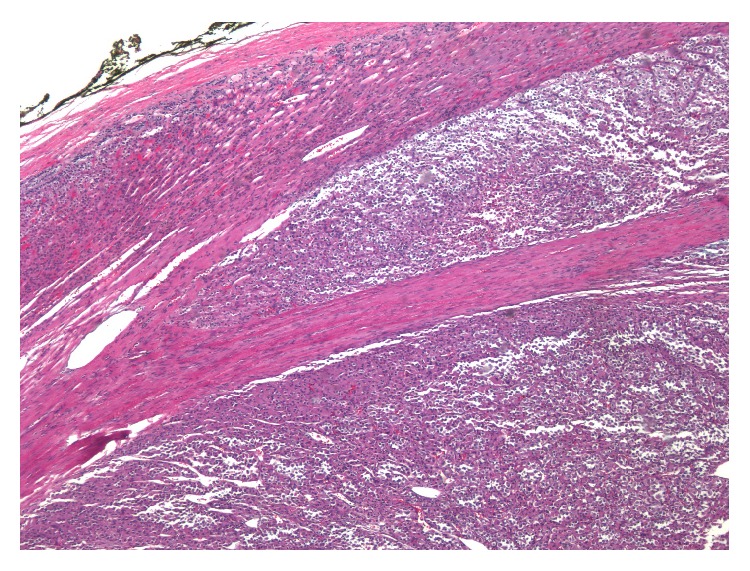
40x magnification: the tumor shows partial invasion through the tumor capsule, indicating at least borderline malignant potential.

**Figure 3:**
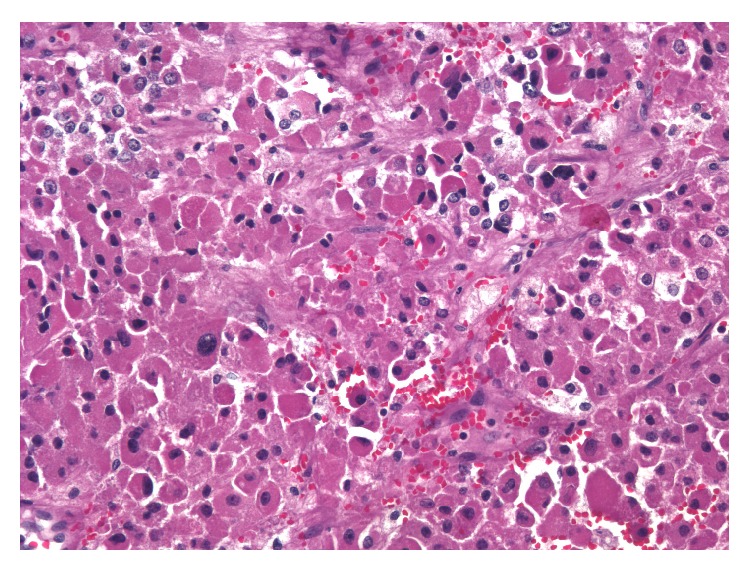
200x magnification: the tumor is composed entirely of large cells with abundant granular eosinophilic cytoplasm and indistinct cellular boundaries. Mild nuclear atypia is seen. Mitotic figures are not evident.

Grossly the mass was found to be a 10.5 cm, pale red-brown variegated mass with areas of hemorrhage and central scarring. Microscopic examination showed clusters of large cells with abundant eosinophilic and granular cytoplasm, consistent with an OAN. The mitotic rare was low (4 per 50 high-power fields) and no atypical mitoses were seen. It was found to invade the capsule (but not beyond) (Figures 2 and 3), and no lymphovascular invasion was identified. With these features, the tumor is best classified as a borderline OAN of uncertain malignant potential.

## 3. Discussion

OAN has been described in only 150 cases in the literature. It typically presents between the ages of 27–72, with a relatively broad age distribution. It occurs in females 2.5 times more frequently and occurs more frequently on the left side [2–4].

Most cases present as an incidental finding on CT or magnetic resonance imaging (MRI), and only approximately 17% of OAN are functional masses [3, 4]. When evaluating a patient with an adrenal incidentaloma, the two most important factors are size and functionality. Size has been shown to be a highly sensitive criterion to rule out adrenocortical carcinoma (ACC); a cutoff of 4 cm was associated with a 90% sensitivity in one series [5].

In addition to size, presence of necrosis [6], absence of fat [7], and chemical shift MRI [8] are other important radiographic metrics used to distinguish benign adenoma from ACC. However, there are no reliable CT or MRI findings that will distinguish OAN from other adenomas [4]. Khan et al. recently published a retrospective review of the imaging findings of 18 patients, in hopes of finding criteria to distinguish benign and malignant OAN. The authors determined that malignant OAN was more likely to be large, lipid-poor, with central necrosis, and lower percentage enhanced washout [9].

It is also important to rule out a functional adenoma, since surgery may be indicated regardless of size [10]. Although rare, there have been reports of OAN mimicking syndromes such as pheochromocytoma [11], aldosteronoma [12], Cushing's syndrome [13], and virilizing tumors [14]. The full battery of biochemical testing should be performed on all patients with incidental masses, regardless of size.

While biopsy and fine-needle aspiration are possible, distinguishing OAN from other adrenal masses using this method can be difficult [15]. Since OAN tend to be larger than other benign adenoma, they usually meet criteria for surgical removal [4]. While open approach is the traditional gold standard, laparoscopic and robotic approaches have been gaining popularity in an attempt to reduce morbidity [7].

On gross pathologic analysis, these masses tend to be large, encapsulated, and well circumscribed. The average diameter is about 8 cm [4]. These tumors are generally brown, yellow, or mahogany, with variable amounts of hemorrhage or necrosis. Microscopically these tumors consist of large cells with abundant eosinophilic, granular, and mitochondria-rich cytoplasm. Cellular membranes are indistinct. The neoplastic cells show sheet-like, trabecular, or papillary growth patterns. Atypia is common, but marked pleomorphism and mitotic figures are rare [4].

Differentiation between benign and malignant forms of OAN can be difficult. The Lin-Weiss-Bisceglia criteria define malignancy by the presence of any one of the following microscopic features: (1) mitotic rate greater than 5 mitotic figures per 50 high-power fields, (2) atypical mitoses, or (3) invasion of venous structures. If no criteria of frank malignancy are present, the minor criteria for borderline malignancy must be assessed. The criteria for tumors of borderline malignant potential include the following: (1) increased size (>10 cm and/or >200 g), (2) necrosis, (3) capsular invasion or (4) sinusoidal invasion. A diagnosis of a benign tumor (oncocytoma) can only be given in the absence of all of the aforementioned criteria for frank or borderline malignancy [16].

In the case of benign OAN, prognosis after surgical resection is considered to be good [4]. If adrenocortical carcinoma is found, adjacent or distant organ involvement at the time of diagnosis is common and associated with a 20–35% 5 year survival [17]. After successful surgical resection of organ-confined disease, a 50–60% 5 year survival can be expected [18]. While adjuvant therapy has been described, there are no specific studies evaluating its use in malignant oncocytic neoplasms.

## 4. Conclusion

We present a case of a 7.2 cm adrenal incidentaloma eventually diagnosed as OAN after surgical removal. OAN is a rare tumor with roughly only 150 cases described in the literature. Routine imagining techniques cannot reliably differentiate OAN from other adrenal adenomas, so the diagnosis is histologic. Microscopically OAN is composed entirely of large cells with abundant eosinophilic and densely granular cytoplasm and indistinct cell borders. Surgical resection is generally curative, except in the case of malignancy, where recurrence is common.

## References

[1] Chang A., Harawi S. J. (1992). Oncocytes, oncocytosis, and oncocytic tumors. *Pathology Annual*.

[2] Kitching P. A., Patel V., Harach H. R. (1999). Adrenocortical oncocytoma. *Journal of Clinical Pathology*.

[3] Wong D. D., Spagnolo D. V., Bisceglia M., Havlat M., McCallum D., Platten M. A. (2011). Oncocytic adrenocortical neoplasms—a clinicopathologic study of 13 new cases emphasizing the importance of their recognition. *Human Pathology*.

[4] Mearini L., Del Sordo R., Costantini E., Nunzi E., Porena M. (2013). Adrenal oncocytic neoplasm: a systematic review. *Urologia Internationalis*.

[5] Angeli A., Osella G., Alì A., Terzolo M. (1997). Adrenal incidentaloma: an overview of clinical and epidemiological data from the National Italian Study Group. *Hormone Research*.

[6] Boland G. W. (2011). Adrenal imaging. *Abdominal Imaging*.

[7] Tirkes T., Gokaslan T., McCrea J. (2011). Oncocytic neoplasms of the adrenal gland. *American Journal of Roentgenology*.

[8] Haider M. A., Ghai S., Jhaveri K., Lockwood G. (2004). Chemical shift MR imaging of hyperattenuating (>10 HU) adrenal masses: does it still have a role?. *Radiology*.

[9] Khan M., Caoili E. M., Davenport M. S. (2014). CT imaging characteristics of oncocytic adrenal neoplasms (OANs): comparison with adrenocortical carcinomas. *Abdominal Imaging*.

[10] Mantero F., Terzolo M., Arnaldi G. (2000). A survey on adrenal incidentaloma in Italy. Study Group on Adrenal Tumors of the Italian Society of Endocrinology. *The Journal of Clinical Endocrinology and Metabolism*.

[11] Li M., Wenig B. M. (2000). Adrenal oncocytic pheochromocytoma. *The American Journal of Surgical Pathology*.

[12] Mete O., Asa S. L. (2009). Aldosterone-producing adrenal cortical adenoma with oncocytic change and cytoplasmic eosinophilic globular inclusions. *Endocrine Pathology*.

[13] Kabayegit O. Y., Soysal D., Oruk G. (2008). Adrenocortical oncocytic neoplasm presenting with Cushing's syndrome: a case report. *Journal of Medical Case Reports*.

[14] Gumy-Pause F., Bongiovanni M., Wildhaber B., Jenkins J. J., Chardot C., Ozsahin H. (2008). Adrenocortical oncocytoma in a child. *Pediatric Blood & Cancer*.

[15] Lee C. K., Choi K. H., Cha Y. J. (2011). Large oncocytic adrenocortical tumor with uncertain malignant potential. *Korean Journal of Urology*.

[16] Lau S. K., Weiss L. M. (2009). The Weiss system for evaluating adrenocortical neoplasms: 25 years later. *Human Pathology*.

[17] Allolio B., Fassnacht M. (2006). Clinical review: adrenocortical carcinoma: clinical update. *The Journal of Clinical Endocrinology and Metabolism*.

[18] Kurek R., Von Knobloch R., Feek U., Heidenreich A., Hofmann R. (2001). Local recurrence of an oncocytic adrenocortical carcinoma with ovary metastasis. *The Journal of Urology*.

